# Assessing the rereading effect of digital reading through eye movements using artificial neural networks

**DOI:** 10.3389/fpsyg.2025.1576247

**Published:** 2025-08-21

**Authors:** Ying Xu, Mingzhen Liang, Yuanyuan Jin, Ligang Wang, Wenbin Gao, Ting Tao

**Affiliations:** ^1^CAS Key Laboratory of Mental Health, Institute of Psychology, Beijing, China; ^2^Department of Psychology, University of Chinese Academy of Sciences, Beijing, China

**Keywords:** rereading effect, digital reading, eye movement, saccade, neural network model

## Abstract

**Objective:**

This study aimed to investigate the differences in eye movement characteristics between first reading and rereading and to develop a neural network model for classifying these reading practices. The primary goal was to enhance the understanding of rereading identification and provide insights into assessing students’ text familiarity.

**Methods:**

We compared eye movement metrics during first reading and rereading, focusing on parameters such as total reading time, fixation duration, regression size, regression count, and local eye movement behaviors within areas of interest (AOIs). Pupil size, the proportion of fixation duration, and regression duration within and across lines were also examined. A neural network model was constructed to classify the reading practices based on these metrics.

**Results:**

During rereading, students exhibited shorter total reading time, fixation durations, and fewer regression counts compared to first reading. Regression size was longer during rereading. Local eye movement behaviors within AOIs were also reduced. However, pupil size, the proportion of fixation duration, and regression duration within and across lines were not useful in identifying rereading. The neural network model achieved an accuracy of 0.769, precision of 0.774, recall of 0.788, and an F1-score of 0.781.

**Conclusion:**

The findings demonstrate distinct eye movement patterns between first reading and rereading, highlighting the effectiveness of certain metrics in differentiating these practices. The neural network model provides a promising tool for rereading identification. These results expand our understanding of rereading behavior and offer valuable insights for assessing students’ text familiarity.

## 1 Introduction

Reading is a necessary cognitive activity in students’ daily life and learning. Reading comprehension skills enhance people’s problem-solving and thinking abilities, playing an important role in their growth and development (Björn et al., 2016; García-Madruga et al., 2016). However, with the development of digital multimedia, the forms of reading have become more diverse, influencing students’ reading behaviors and performance, shaping a preference for digital devices rather than paper books (Wolf and Potter, 2018; Yusof, 2021). As such, digital reading offers portability, personalized settings, and interactive features, making it increasingly popular among learners (Schwabe et al., 2022; Liao et al., 2024; Peras et al., 2023; Singer Trakhman and Alexander, 2017; William et al., 2025; Yusof, 2021).

In the context of the widespread use of digital reading, it is crucial to develop good reading habits and master effective reading strategies. Among them, rereading is widely recognized as the most commonly used reading comprehension strategy (Hyönä and Niemi, 1990). Reading the text multiple times is an intuitive reading technique readers use when they do not understand the content they have just read. Rereading can help students read more quickly, better memorize details, deepen their comprehension of the text, and improve reading efficiency (Margolin and Snyder, 2018; Xue et al., 2020). The phenomenon of improved reading performance is referred to as the “rereading effect” or “rereading benefit,” which has received considerable attention in many studies (Bromage and Mayer, 1986; Raney, 2003; Callender and McDaniel, 2009; Kuijpers and Hakemulder, 2018; Rawson et al., 2000; Seban et al., 2025). The rereading effect could be explained by the hypothesis proposed by Hyönä and Niemi (1990) that the first reading creates a mental representation in the reader’s mind, and rereading can activate this representation to facilitate easier understanding. Similarly, van Moort et al. (2021) believed the prerequisite for constructing coherent and accurate mental representations is to continuously validate contextual information and background knowledge through interaction, leading to better reading comprehension and performance.

Besides an intuitive and widely used technique to understand the content thereof, rereading has also served as a typical paradigm by researchers to investigate the rereading effect and its limiting factors (Margolin and Snyder, 2018; Xue et al., 2020).

To be specific, Margolin and Snyder (2018) found that a rereading strategy significantly improved overall meta-comprehension and comprehension ability of relevant and negation information. Xue et al.'s (2020) study indicated that readers’ accuracy in identifying the main theme of an article increased after rereading. This supports studies confirming rereading as a useful strategy for improving overall comprehension (Rawson et al., 2000). As the number of reading sessions increases, readers become more familiar with the text, reading speed increases, and more details are remembered, deepening their understanding of the article (Kamienkowski et al., 2018; Raney, 2003).

However, the rereading effect can be limited by many factors, including reading level, fatigue, and text complexity (Kuijpers and Hakemulder, 2017; Callender and McDaniel, 2009). The specific rereading effect is influenced incredibly by the reader’s reading proficiency, which has been confirmed by a number of existing studies. It has been found that higher reading proficiency may benefit from the rereading effect more.

For example, in the study of Callender and McDaniel (2009), the reading proficiency of participants was measured by additional reading tests, and results revealed that rereading was more beneficial for high-level readers, who demonstrated more significant effects thereof than lower-level readers. These findings echo those of earlier studies. In a study by Barnett and Seefeldt (1989), according to their ACT scores, participants were divided into two different groups. It has been found that the rereading effect benefited high-ability readers more by improving deeper reading comprehension and cognitive representation, while low-ability readers mainly improved surface information processing through rereading. However, in some studies, it is readers who have decoding and reading efficiency problems that benefit more from the rereading effect, contrary to previous arguments. A significant interaction has been found between reading ability and rereading times during a short-answer reading test, with low-ability readers being more aware of the benefits of rereading than high-ability readers (Callender and McDaniel, 2009). The impact of rereading benefits for readers with varying reading proficiency remains unclear, indicating the need for further research.

By recording directly the cognitive processes during reading, eye-tracking technology captures the eye movement trajectory without interfering with participants’ reading process, compensating for the inability of traditional post-event assessment methods and providing behavioral information for a better investigation of the rereading effect. Especially in natural online reading situations, eye-tracking technology helps sensitively and effectively record online reading behavior, indicating the different cognitive processes between first reading and rereading. Numerous studies have identified mappings between eye movement indicators and the rereading effect, mainly reflected in the reduced total reading time, decreased regression time, fewer regressions, and increased skipping rates (Hyönä and Niemi, 1990; Kaakinen and Hyönä, 2007; Raney, 2003). In the study by Copeland and Gedeon (2013), it was found that the number and duration of fixations of typically-developed university students are related to the familiarity of the text. The more familiar the vocabulary, the fewer the number and duration of fixations. Furthermore, during the second reading, not only were the duration and number of fixations reduced, but fewer regressions may indicate a greater contextual effect. These differences in eye-tracking metrics reveal the underlying mechanisms of the rereading effect. By analyzing the eye movement pattern of English native speakers without a professional literacy training background, Xue et al. (2020) confirmed that rereading enhances text reading fluency, as evidenced by reduced regression time and total reading time, which are characteristic of later stages in the reading and comprehension process. In the final reading, the skipping rate was also higher, indicating a lower probability of fixating on any given word. However, rereading appears not to affect eye movement measures during the early stages of reading processing. For instance, in the study, the first fixation and gaze durations during rereading were similar to those during the initial reading (Xue et al., 2020). Further research on eye movements during rereading is essential for identifying reliable indicators of these movements and enhancing our understanding of the cognitive processes involved in rereading.

Rereading eye-tracking studies typically involves analyzing differences in eye-movement patterns between the first and second readings to understand how readers process and comprehend text. Artificial neural networks (ANNs) are mathematical models based on the fundamental principles of biological neural networks, simulating the complex information processing mechanisms of the human brain’s neural system (Basheer and Hajmeer, 2000). They are adaptive, non-linear dynamic systems. In recent years, an increasing number of scholars have attempted to combine neural network classification algorithms with eye-tracking research on reading, achieving promising results (Aracena et al., 2015; Bellet et al., 2019; Frey et al., 2021; Kastrati et al., 2021; Lim et al., 2022; Liu et al., 2021; Rakhmatulin and Duchowski, 2020). Compared to traditional methods, neural network approaches offer significant advantages in natural reading studies, as the various psycholinguistic and contextual text features, as well as cognitive features such as readers’ eye movements, exhibit multiple potential linear and nonlinear relationships. Neural network algorithms often yield optimal results in classification and prediction tasks (Jacobs and Kinder, 2018). For example, Xue et al. (2019) found neural network methods to be a highly effective approach for predicting two eye-tracking parameters (total reading time and fixation probability) using seven surface features. Additionally, neural networks offer improvements over traditional methods in improving the predictive power of eye movement feature indicators for unseen texts (Wang and Zhao, 2019), addressing conceptual ambiguities in existing digital literacy assessments, and increasing the objectivity and effectiveness of assessment metrics. Based on previous research, neural network models currently demonstrate promising performance in the field of reading eye movements, but some issues remain. First, there is currently a lack of neural network models related to rereading effects. Additionally, eye movement metric-based classification models constructed using data-driven methods often exhibit good fit on training datasets but struggle to generalise to other datasets. Considering model transferability, it is necessary to select specific eye movement metrics based on knowledge-driven approaches for model construction. For example, eye movements such as regressions are important indicators of rereading effects (Hyönä and Niemi, 1990; Kaakinen and Hyönä, 2007; Raney, 2003). A classification model for rereading can be constructed based on the time series analysis of eye movements, thereby enhancing the model’s interpretability.

The goal of this study is to investigate the differences in eye movement patterns between initial readings and rereadings during discourse comprehension, with an emphasis on identifying key eye-tracking indicators associated with rereading. The current study focuses on three main research objectives. First, it examines how reading proficiency influences the rereading effect by analyzing the rereading behaviors of students at various reading levels, thereby providing empirical evidence regarding the impact of reading ability on text rereading. Second, by utilizing eye-tracking technology, this study aims to uncover potential eye movement indicators of rereading and to explore the relationship between these eye movements and cognitive processing during rereading. This will contribute to identifying reliable indicators of eye movements that represent the rereading process and deepen our understanding of the cognitive mechanisms involved. Lastly, the study employs the identified eye movement indicators for rereading to predict the outcomes of first readings and rereadings by developing neural network prediction models. This approach optimizes the use of eye movement indicators and enhances their practical value in real-world reading scenarios. To address these objectives, the following specific research questions are proposed:

1. How does reading proficiency level influence the rereading effect?
2. How does reading proficiency level influence the pattern of eye movements during rereading compared to initial reading, and what specific eye-tracking indicators reliably differentiate rereading from initial reading?
3. Can neural network models effectively predict reading outcomes (e.g., comprehension) for both initial readings and rereadings using the identified eye movement indicators associated with rereading?

## 2 Methodology

### 2.1 Participants

This study employed convenience sampling, with recruitment announcements posted online and offline at the university and nearby universities. In total, 107 native speakers of Chinese volunteered to participate in the study. Due to technical issues with the eye tracker at the start of the research, the data of 4 participants were of insufficient quality and could not be analyzed. Thus, 103 participants were included in the analysis. They were aged 18–32 years (M ± SD = 23.17 ± 3.00) and included 41 males and 62 females. For more details, please refer to the Supplementary materials. They all participated in the College English Test (CET) Band 4 (M ± SD = 522.65 ± 65.29) and Band 6 (M ± SD = 493.72 ± 61.26) in China. Based on the results of the CET-4 and CET-6 exams, reading proficiency was categorized into high and low groups (F_CET-4(1, 101)_ = 195.761, *p* < 0.001; F_CET-6(1, 101)_ = 159.732, *p* < 0.001), with 40 participants in the high-level group and 63 in the low-level group. After normality tests, both the CET-4 and CET-6 scores were found to follow a normal distribution (CET-4 scores: *p* = 0.132; CET-6 scores: *p* = 0.200).

All participants had normal or corrected-to-normal eyesight, and none had been diagnosed with a reading or learning disability. Participants provided written informed consent before testing and were paid for participating.

### 2.2 Apparatus

Eye movements were recorded with an Eye Link 1,000 eye-tracker (SR Research Ltd., Canada). The sampling frequency was 1,000 Hz. Participants’ heads were kept still using a chin-and-head rest. Stimuli were presented on a 27-inch HP monitor with a resolution of 2,540 × 1,440 pixels and a refreshment rate of 165 Hz, 71 cm away from the reader. The monitor dimensions were 614 mm in length and 512 mm in width. The text area had margins of 200 mm at the top and bottom, and 300 mm on the left and right. The text materials were programmed and presented using Eye-link Experiment Builder (EB) software. The font of the text was Courier New, with a font size of 16 points (21 pixels) and a line spacing of 2.5. The visual angle of each letter was 0.41° horizontally. Prior to each reading session, a standard 9-point eye calibration was performed to ensure an average spatial resolution error of less than 0.5° of the angle of vision.

### 2.3 Materials

The text materials for the experiment were selected from articles in the 2021–2022 College English Test (CET) Level 6, with seven articles of different topics and genres initially chosen. Served as the pilot study, eleven undergraduate or graduate students (not participating in the formal experiment) were asked to provide a summary of the main ideas and rate the difficulty level of all seven articles from 1 (very simple) to 5 (very difficult). An article with a moderate level of difficulty was chosen for the formal experiment based on their feedback.

The main topic of the selected article is campus lending issues. There are four paragraphs. The introductory paragraph poses the research question. The two middle paragraphs present the main viewpoints and information. The concluding paragraph summarizes and elevates the main idea of the article. The article was modified by removing detailed questions that required memorization and retaining comprehension questions. The resulting text had 315 words, and there were 4 multiple-choice questions with 4 answer options each. Participants received one point for each correct answer and no points for incorrect answers. An example of a question is as follows:

1. *What do we learn from the policy of forgiving student debt?*

- *It has benefited both the economy and the underprivileged.*
- *Canceling student debt benefits wealthy families the most.*
- *Forgiving student debt provides little benefit to universities.*
- *Low-income families owe the biggest amount of student debt.*

The content of the passage was the same for both the first reading and rereading. However, to ensure that participants reread the article carefully from the beginning, the order of the comprehension questions was changed.

### 2.4 Procedure

1. Participants were introduced to the eye-tracking laboratory and familiarized with the experimental environment and equipment to alleviate any anxiety or fear. They were then asked to complete a demographic questionnaire to provide basic information.
2. The experimenter explained the purpose of the study, experimental procedures, and instructions to the participants, stating:
  “Hello, thank you for participating in this study. We are conducting eye-tracking research related to English reading. Prior to the experiment, we will calibrate your eyes, and then present English reading materials on the screen. Please read the article carefully at your daily reading speed and habits. There is no time limit for reading, and you should focus on understanding the content. After reading, please press the space bar to proceed to the following four multiple-choice questions. Throughout the experiment, please keep your eyes on the screen.”
3. After the first reading and comprehension task, participants were asked to rate the perceived difficulty of the article from 1 (very simple) to 5 (very difficult).
4. They were then given a three-minute break and asked if they were ready to proceed to the next task.
5. Prior to the rereading task, a nine-point calibration of eye movements was conducted. Participants were then instructed to reread the article and complete the following four multiple-choice questions again, although the content was the same as in the first article. To ensure that participants read the article carefully during the subsequent rereading, they were instructed to treat it as a new article and read it from the beginning. They were told that this was *“because the comprehension questions presented later differed from those in the first reading.”* In fact, only the presentation order of the reading comprehension questions was manipulated, while the content of the questions remained unchanged.
6. After the rereading and comprehension tasks, participants were again asked to rate the perceived difficulty of the article from 1 (very simple) to 5 (very difficult).

**Figure 1:**
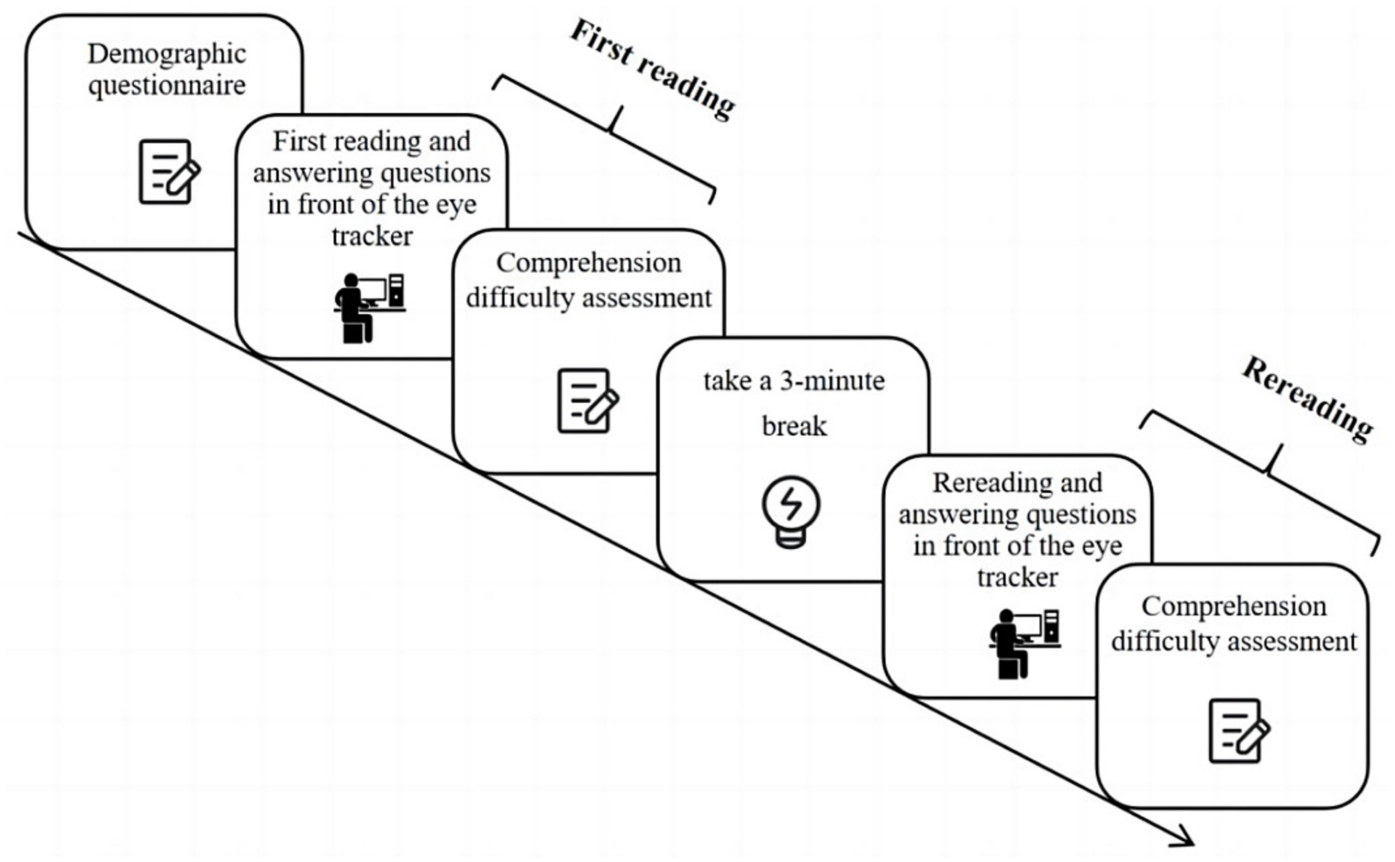
The experiment procedure.

Their eye movements were recorded throughout the reading and comprehension tasks, but only the eye-tracking data related to discourse reading were utilized in this study. Altogether, the experiment took about 30 min (see Figure 1 for an illustration of the procedure).

### 2.5 Data analysis

#### 2.5.1 Ratings

Descriptive statistics were used to analyze the demographic variables, reading proficiency, reading comprehension scores, and comprehension difficulty ratings. Paired-sample t-tests were performed to examine the differences between the two readings in terms of comprehension scores and comprehension difficulty ratings.

#### 2.5.2 Analysis of eye tracking parameters

1. Data preprocessing: Events outside the text area were excluded. Fixations with a duration of less than 80 ms were merged into the fixation points within the visual angle of one letter (0.41°) of the adjacent letter. Fixation points with a duration of less than 80 but greater than 1,200 ms were removed. Events within 100 ms before and after a blink were excluded. In total, 6.6% of the fixation points were deleted during the first reading, and 7.9% during the rereading.
  This experiment adopted a 2 (reading proficiency: high vs. low) × 2 (reading frequency: first reading vs. rereading) factorial mixed design. The independent variables were reading proficiency, which is a between-group variable, and reading frequency, a within-group variable. The dependent variables were reading performance and various eye movement measures.
2. Grouping: Based on previous research, reading proficiency may affect the primacy effect. Therefore, in this study, participants were randomly selected, and their Band 4 and Band 6 scores were both normally distributed (Band 4: *p* = 0.132, Band 6: *p* = 0.200) to eliminate the influence of differences in reading proficiency. In addition, reading proficiency was included as an independent variable to test its controlling effect on the results. Specifically, based on the College English Test (CET) Band 4 and Band 6 scores, participants were divided into two groups using cluster analysis, namely the high and low reading proficiency groups. The high proficiency group consisted of 40 participants, and the low proficiency group of 63.
3. A repeated-measures analysis of variance (ANOVA) was conducted to examine the differences in reading and eye movement measures between readers with different levels of reading proficiency during the first reading and rereading. Eye movement measures were categorized into global and local measures. Global measures were based on the area of interest (AOI) of the entire rectangle text area, including saccade, regression, and fixation behaviors.
4. Local AOI was identified by 11 participants who extracted the core words and phrases essential to understanding the article. AOIs selected six times or more were used together as local AOIs at the lexical level. The parameters for local AOIs included total fixation behaviors, regression behaviors, and first and second fixation or reading behaviors.

### 2.6 Neural network modeling

This study used a feedforward neural network to identify and analyze reading eye movement data, and constructed prediction models for the first reading and rereading. The neural network model was developed using Keras in Python (Chollet and others, 2015). We selected eye movement parameters with significant main effects of reading frequency as the sample data for the input layer. The eye movement parameters were normalized to a range between 0 and 1 when entering the input layer. The output layer comprised two neurons divided into two categories: “first reading” and “rereading.” Five-fold cross-validation was employed to reduce over fitting and to render the model’s performance less sensitive to the data partition. Besides, this study adopted the following evaluation metrics to assess predictor performance: accuracy, precision, recall, F_1_-score, and cross-entropy error (loss). The accuracy, precision, recall, and F_1_-score relate to true positives (TP), false positives (FP), true negatives (TN), and false negatives (FN) as:

1. *precision* (*p*) = TP/(TP + FP).
2. *recall* (*r*) = TP/(TP + FN).
3. *accuracy* (*a*) = (TP + TN)/(TP + FP + FN + TN).
4. *F_1_-score* = 2*p*r/(*p* + *r*).

Here, cross entropy error (loss) is a commonly used loss function used to measure the difference between the predicted and actual results of the model. Furthermore, in neural networks, Feature Importance (FI) is a valuable tool for evaluating the significance of input features in model prediction (Strobl et al., 2009). This study utilized Permutation Importance, a method that randomly disrupts a single feature to observe the impact on model accuracy, thereby obtaining a ranking of FI.

## 3 Results

### 3.1 Results of rereading effect and reading proficiency

Table 1 shows the descriptive statistics and results of the t-test of comprehension difficulty and reading comprehension scores in the first reading and rereading. There was a significant difference (*p* < 0.001) in the comprehension difficulty ratings and reading comprehension scores of participants between the first reading and rereading. After rereading the article, participants reported a significant decrease in the perceived difficulty of the article and a significant improvement in their comprehension scores.

**Table 1:** Descriptive statistics and results of the paired *t*-test of comprehension difficulty and reading comprehension scores (*N* = 103).

Type of scores	First reading (M ± SD)	Rereading (M ± SD)	*t*	*p*
Comprehension difficulty	3.408 ± 0.954	3.165 ± 1.011	3.800	< 0.001
Reading comprehension scores	0.393 ± 0.279	0.498 ± 0.288	−4.06	< 0.001

To examine the effects of different reading levels and rereading strategies on reading comprehension performance, we employed a two-factor mixed design analysis of variance (ANOVA). The results of the ANOVA revealed a significant main effect of reading level on reading comprehension scores (F_(1, 101)_ = 10.21, *p* < 0.05, η^2^p = 0.009), with the high-level group achieving higher scores; the main effect of rereading was also significant (F_(1, 101)_ = 14.96, *p* < 0.05, η^2^p = 0.13), with rereading resulting in significantly higher reading comprehension scores than the first reading. No significant interaction effect was found between the two factors (*p* > 0.05). Similarly, significant main effects of rereading and reading level were found in article difficulty ratings (F_(1, 101)_ = 16.85, *p* < 0.05, η^2^p = 0.14; F_(1, 101)_ = 8.56, *p* < 0.05, η^2^p = 0.08). High-level readers rated article difficulty lower, and the difficulty ratings during rereading were significantly lower than those during the first reading. The interaction between English proficiency and rereading was nearly significant (F_(1, 101)_ = 2.77, *p* = 0.09, η^2^p = 0.03). Further simple effect analysis revealed that the rereading effect was more pronounced in the high-level group. Specifically, the difficulty ratings of the high-level group decreased significantly upon rereading (*p* < 0.05), while the difference in difficulty ratings between the two readings was not significant in the low-level group (*p* > 0.05), indicating that readers in the high-level group benefited more from rereading.

### 3.2 Results of eye movement parameters between the first reading and rereading

The descriptive analyses of participants’ eye movements during the first reading and rereading, categorized by low- and high-level reading proficiency, are presented in the Supplementary materials. To examine the effects of reading frequency and reading proficiency on eye movement behaviors, we evaluated the eye movement parameters globally and locally.

#### 3.2.1 Global measures

**Figure 2:**
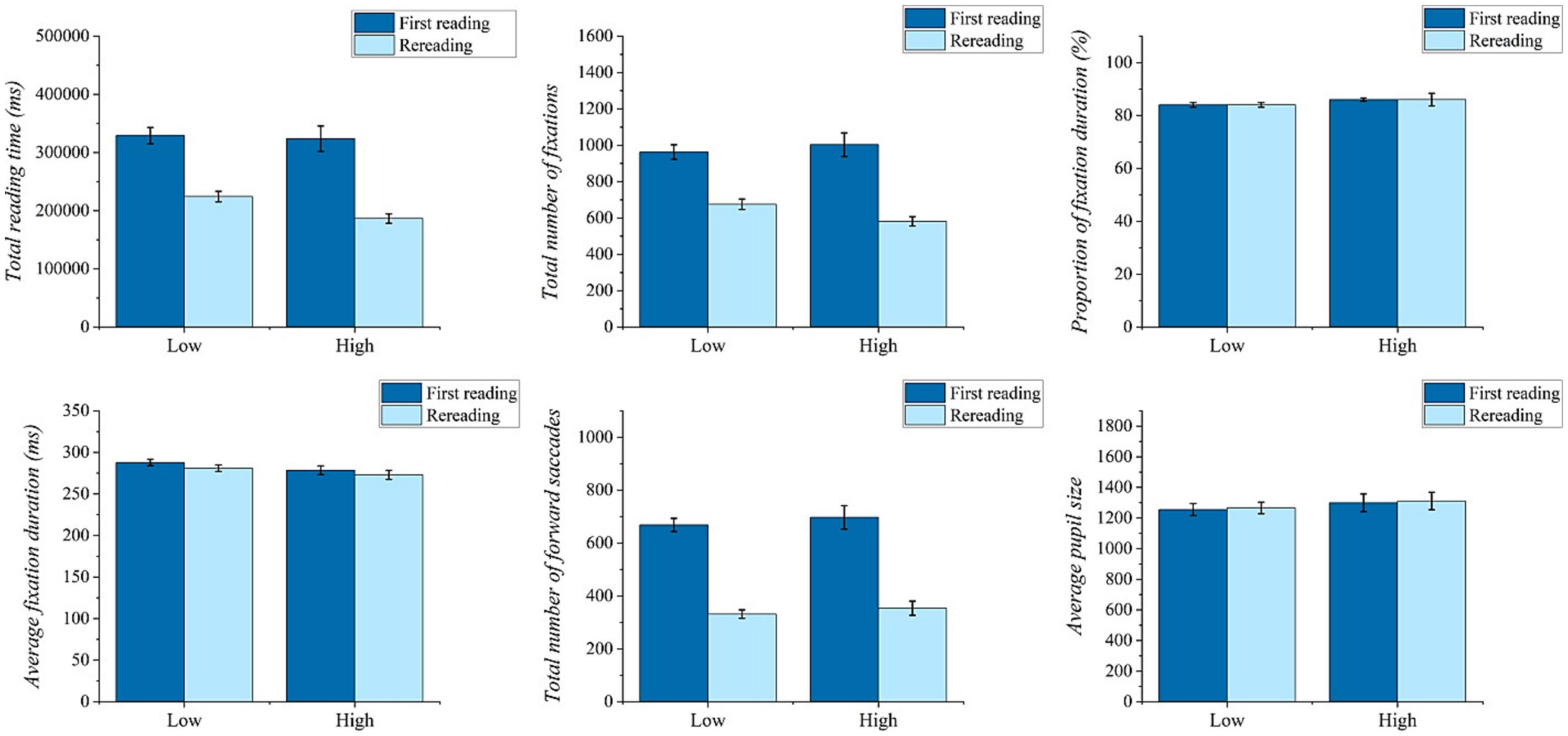
Bar chart of global eye movement measures in the repeated measures analysis of variance (ANOVA). Error bar is constructed using one standard error from the mean.

As illustrated in Figure 2, aside from the proportion of fixation duration and average pupil size, there are significant main effects of reading frequency on total reading time (*F*_(1,101)_ = 107.25, *p* < 0.001, η^2^_p_ = 0.515), total number of fixations (*F*_(1,101)_ = 103.83, *p* < 0.001, η^2^_p_ = 0.515), average fixation duration (*F*_(1,101)_ = 17.22, *p* < 0.001, η^2^_p_ = 0.146) and total number of forward saccades (*F*_(1,101)_ = 476.69, *p* < 0.001, η^2^_p_ = 0.825). After rereading, both total reading time and average fixation duration were significantly shorter than during the first reading. Similarly, the total number of fixations and total number of forward saccades were significantly fewer after rereading compared to those of the first reading. However, the main effect of reading proficiency and interaction effect of reading frequency*reading proficiency were not significant (*p* > 0.05).

In Supplementary Figure S1, except for the average regression durations within line and across lines, there were significant main effects of reading frequency on average regression size (*F*_(1,101)_ = 7.77, *p* < 0.01, η^2^_p_ = 0.071), total regression counts (*F*_(1,101)_ = 82.74, *p* < 0.001, η^2^_p_ = 0.450), average regression distance within line (*F*_(1,101)_ = 9.27, *p* < 0.01, η^2^_p_ = 0.084), average regression counts within line (*F*_(1,101)_ = 90.97, *p* < 0.001, η^2^_p_ = 0.474), average regression counts across lines (*F*_(1,101)_ = 45.83, *p* < 0.001, η^2^_p_ = 0.312), and average regression distance across lines (*F*_(1,101)_ = 11.54, *p* < 0.001, η^2^_p_ = 0.103). After rereading, the average regression size and average regression distance within the line significantly increased compared to the first reading, while the average regression distance across lines significantly decreased. The average regression counts within the line and across lines were significantly lower after rereading compared to the first reading. There were no significant main effects of reading proficiency on global eye movement measures (*p* > 0.05). Excluding the average regression counts within the line (*F*_(1,101)_ = 4.13, *p* < 0.05, η^2^_p_ = 0.039), there were no significant interaction effects between reading frequency and reading proficiency on other global eye movement measures (*p* > 0.05).

#### 3.2.2 Local measures

Likewise, Supplementary Figure S2 shows the results for the significant main effects of reading frequency on all local eye movement measures. After rereading, the IA_Total fixation duration and IA_Average selective regression path duration were significantly shorter compared to the first reading, while IA_Total number of fixations and IA_Average regression out counts were significantly lower. Aside from the IA_Average regression out counts (*F*_(1,101)_ = 4.34, *p* < 0.05, η^2^_p_ = 0.041) and IA_Average selective regression path duration (*F*_(1,101)_ = 6.41, *p* < 0.05, η^2^_p_ = 0.060), there were no significant main effects of reading proficiency on other local measures (*p* > 0.05). Readers with lower reading proficiency had significantly higher IA_Average regression out counts and a significantly shorter IA_Average selective regression path duration than readers with higher reading proficiency. Except for IA_Average regression out counts (*F*_(1,101)_ = 4.60, *p* < 0.05, η^2^_p_ = 0.044) and the IA_Average selective regression path duration (*F*_(1,101)_ = 4.38, *p* < 0.05, η^2^_p_ = 0.042), there were no significant interaction effects between reading frequency and reading proficiency on other local eye movement measures (*p* > 0.05).

Finally, Supplementary Figure S3 gives the results for the significant main effects of reading frequency on all first-pass and second-pass local eye movement measures. After rereading, the IA_Average first fixation duration, IA_Average first-pass reading time, IA_Average second fixation duration, and IA_Average second-pass reading time were significantly shorter compared to the first reading. IA_Average first fixation counts and IA_Average second fixation counts were significantly lower after rereading compared to the first reading. Except for the IA_Average first-pass reading time (*F*_(1,101)_ = 5.11, *p* < 0.05, η^2^_p_ = 0.048) and IA_Average first fixation counts (*F*_(1,101)_ = 4.90, *p* < 0.05, η^2^_p_ = 0.046), there were no significant main effects of reading proficiency on other first-pass and second-pass local measures (*p* > 0.05). Readers with lower reading proficiency had significantly longer IA_Average first-pass reading time and significantly higher IA_Average first fixation counts compared to readers with higher reading proficiency. Apart from IA_Average first fixation counts (*F*_(1,101)_ = 4.44, *p* < 0.05, η^2^_p_ = 0.042) and IA_Average second fixation duration (*F*_(1,101)_ = 6.99, *p* < 0.05, η^2^_p_ = 0.065), there were no significant interaction effects between reading frequency and reading proficiency on other first-pass and second-pass local measures (*p* > 0.05).

### 3.3 Heat map for first reading and rereading

**Figure 3:**
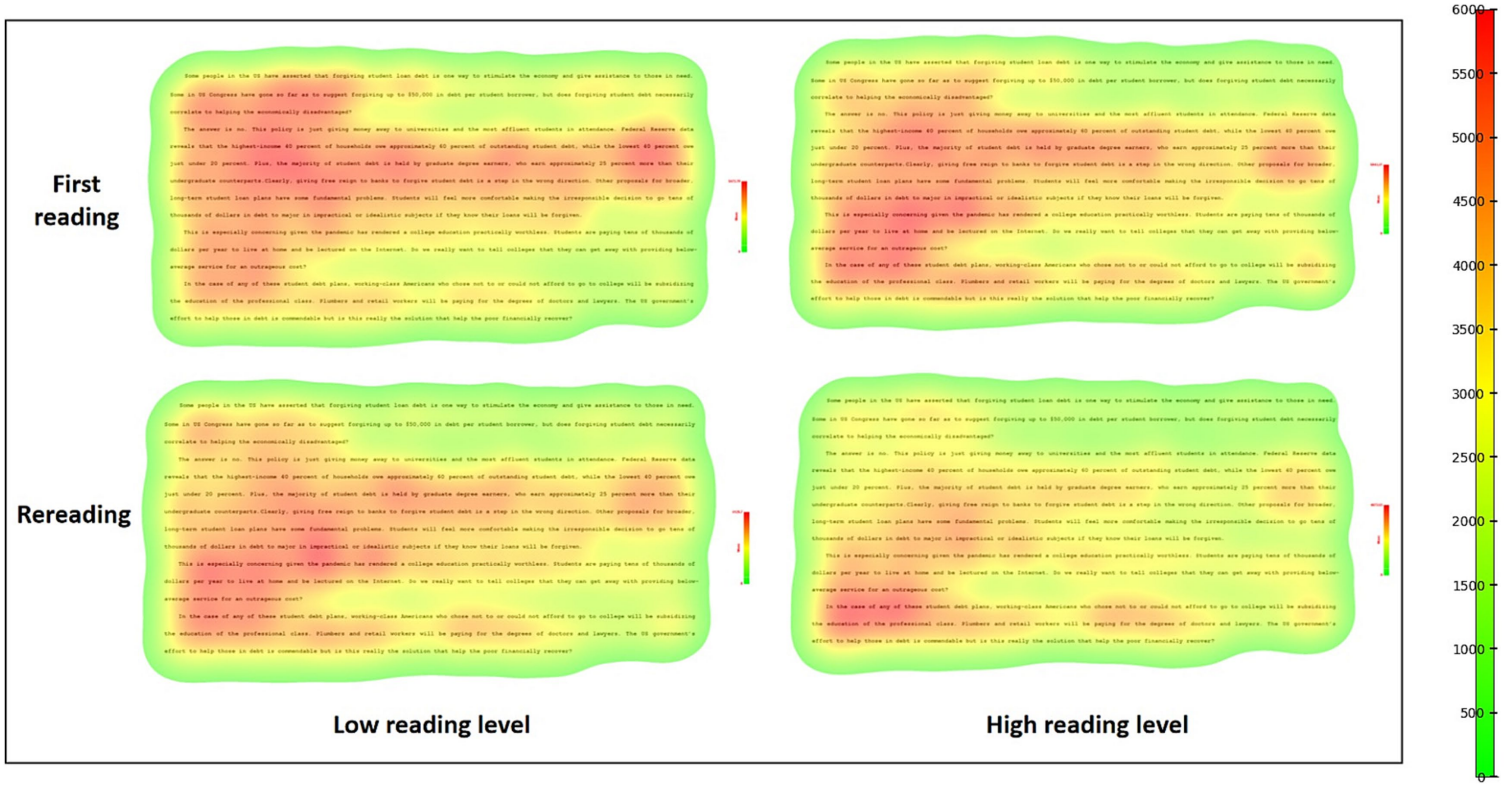
Eye movement heat map of readers with different reading levels during the first reading and rereading.

The heat map is a visual analysis method to mark and display the AOI according to the differences in fixation counts, fixation duration, and viewing position, which is generally marked by color depth, point density, and presentation proportion (Yan et al., 2013). This study focused on the first reading and rereading of students with different reading levels. The specific statistics have been provided in Table 2, and the results are shown in Figure 3.

**Table 2:** Descriptive statistics of the local eye movement parameters (*n*_1_ = 63, *n*_2_ = 40).

Eye movement parameters	Reading proficiency	First reading (ms)		Rereading (ms)	
		M	SD	M	SD
IA_Total fixation duration	Low	276644.508	94695.630	188821.190	64616.970
	High	278509.925	117905.585	157987.975	45294.460
IA_Total number of fixations	Low	962.698	316.921	675.476	229.623
	High	1002.950	408.280	581.675	160.878

Regarding gaze position, participants exhibited a broader range of gaze during their first reading, with specific emphasis on the introductory and middle paragraphs of the article, forming chunk-like hotspots of visual attention. However, a reduced density of fixation is evident in the first and middle paragraphs during rereading, with an increased density of attention in the concluding section. Furthermore, the participants tended to pay more attention to the parts of the text that contain key information when rereading.

Regarding gaze content, during the first reading, readers primarily focused on numerical information, predicate verbs, adjective and adverb phrases, and key noun phrases that aid in comprehending the content, such as “*economically disadvantaged,*” “*highest-income 40 percent,*” “*approximately 60 percent,*” “*concerning,*” and “*student belt.*” After rereading, readers’ attention became more focused on understanding challenging content, such as “*impractical,*” “*idealistic,*” and “*pandemic.*”

### 3.4 Results of predictive modeling

We built a feedforward neural network model with two layers to predict the first reading and rereading. We selected and analyzed the data of 20 eye movement indicators from 103 valid subjects. The 20 eye movement parameters illustrate the significant main effects of reading frequency (see Table 3). Hence, this study chose these parameter groups to classify the first reading and rereading shown in our final model. The middle layer was finally set with 10 neurons.

**Table 3:** Parameter groups and specific parameters for each group.

Parameter group	Eye movement behaviors	Parameters
Global measures	Total gaze behaviors	Total reading time (TRT) Total number of fixations (TNF) Average fixation duration (AFD) Total number of forward saccades (TNFS)
	Regression behaviors	Total regression counts (TRC) Average regression size (ARS) Average regression distance within line (RDWL) Average regression counts within line (RCWL) Average regression distance across lines (RDAL) Average regression counts across lines (RCAL)
Local measures in AOIs	Total gaze behaviors	Total fixation duration (IA_TFD) Total number of fixations (IA_TNF) Average regression out counts (IA_ROC) Average selective regression path duration (IA_SRPD)
	First and second fixation or reading behaviors	Average first fixation duration (IA_FFD) Average first-pass reading time (IA_FRT) Average first fixation counts (IA_FFC) Average second fixation duration (IA_SFD) Average second-pass reading time (IA_SRT) Average second fixation counts (IA_SFC)

The stratified sampling method was used to partition the dataset. Employing randomized five-fold cross-validation, this model obtained an average accuracy of 0.74. The recall, precision, accuracy, F_1_-score and loss of this eye movement parameters predictive model are 0.788, 0.774, 0.769, 0.781, and 0.548, respectively.

As depicted in Supplementary Figure S4, the training set demonstrates a comparable stability with a loss score of approximately 0.525, while the test set maintains a similar stability at around 0.55. Regarding accuracy scores, the training set exhibits a stable performance at approximately 0.74, while the test set shows a consistent stability at around 0.77.

**Figure 4:**
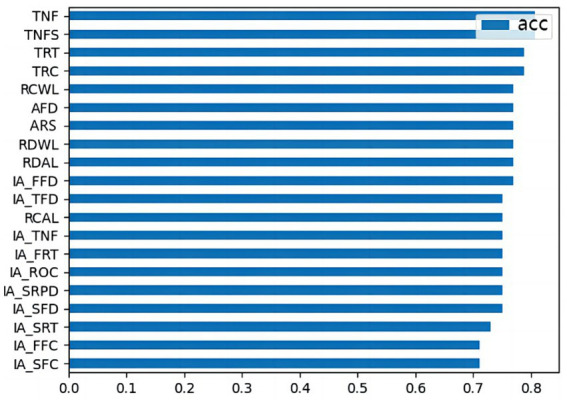
Permutation importance of eye movement parameters.

Figure 4 indicates that the feature importance (FI) of accuracy (acc) ranges between 0.7 and 0.8. It can be observed that removing any individual parameter has minimal impact on the overall predictive performance of the model. Specifically, the FI of local eye movement parameters is higher, while the global eye movement parameters demonstrate relatively lower significance. The top three most important parameters are IA_SFC, IA_FFC, and IA_SRT, whereas the bottom four parameters are TNT, TNFS, TRT, and TRC.

## 4 Discussion

In this study, we compared the differences in the eye movement parameters (including global measures and local measures) and distribution of fixations between the first reading and rereading. We found that rereading had fewer global gaze and regression behaviors, and fewer local eye movement behaviors in AOIs than in the first reading. The distribution of fixations during rereading was more focused on what was considered effective information than the first reading. Moreover, the neural network model performed well, demonstrating that eye-tracking data could be a promising path toward rereading detection.

### 4.1 Rereading effect in different reading levels

In this study, we assessed the rereading effect of students at different reading levels. We set up questions for subjects to assess reading difficulty and questions to examine how well subjects read and understood the text, and in the overall measure, we found a significant main effect of rereading, with subjects’ reading comprehension scores increasing significantly between the first and the rereadings, and comprehension difficulty decreasing significantly between the two readings before and after the rereadings. Besides, our study found differences in the performance of the rereading effect at different reading levels.

In our findings, we observed that the rereading effect positively impacted reading comprehension scores for participants across various reading levels, showing no significant difference between the groups. However, high-level readers derived greater benefit from rereading, particularly concerning their perceived difficulty ratings. Specifically, they reported a notable decrease in the perceived difficulty of the article during the second reading, while the low-level group’s difficulty ratings remained consistent between the two readings. According to landscape theory (van den Broek et al., 1999), both reader ability and the availability of cognitive resources influence the construction of text comprehension, which also explains our current experimental results. High-level readers possess richer English-related background knowledge, enabling them to more efficiently activate connections between textual elements (e.g., causal and anaphoric relationships), thereby maintaining higher standards of coherence. They actively employ constructionist processes to fill information gaps (e.g., generating inferences and integrating background knowledge). In contrast, low-level readers, due to their limited English-related knowledge and background, may need to divert part of their cognitive processing resources to extracting English knowledge (e.g., word recognition) during text processing. As a result, their text comprehension process primarily remains at the memory-based processing level, making it difficult for them to actively construct deeper connections. Our results found that reading proficiency moderates the rereading effect primarily in terms of difficulty scores rather than reading comprehension, which is inconsistent with previous research findings. This may be since most previous studies on the rereading effect have been conducted on native readers, who typically experience few word recognition problems and generally master complex syntactic structures from the outset of their reading (Callender and McDaniel, 2009; Kuijpers and Hakemulder, 2018; Rawson et al., 2000).

However, the subjects in this study were ordinary college students who take English as second language. For them, English is an unfamiliar language compared to their native language, and they are prone to word recognition difficulties and word-level and text-level transfer difficulties during English discourse reading, leading to slow reading or reading behaviors that repeatedly reread the text as a means of having sufficient working memory resources to develop coherent mental representations of the text (Thomas and Healy, 2012). This inefficient approach to word recognition and low-level language processing delays high-level comprehension processes, increases the load on working memory, and ultimately reduces readers’ reading comprehension (Koda, 2007). Thus, in this case, the eye-movement behavior of foreign language readers with high and low reading levels does not differ greatly, requiring similar reading time to be spent, producing similar reading eye-movement processes in terms of gaze duration, number of eye-hopping bouts, return distance, and number of return bouts.

In summary, our study supports the landscape theory and finds that the influence of reading level on the rereading effect may be affected by the language proficiency of the participants. For Chinese university students whose native language is Mandarin, the influence of reading level on the rereading effect is mainly reflected in the perception of difficulty, rather than in the results of reading comprehension.

### 4.2 First reading-rereading differences in global eye movement parameters

In this study, we investigated whether we could assess students’ first reading and rereading via eye-tracking. For the global measures, rereading had a shorter total reading time and average fixation duration, as well as the lower total number of fixations and forward saccades.

These results indicated that rereading digital discourse enhanced readers’ familiarity and understanding of the text content. Specifically, the reduction in fixation duration indicated that readers did not process the coherent information of details during the second reading, which is consistent with the decrease in difficulty scores from high-level readers, indicating more efficient utilization of the built model. As reported by Hyönä and Niemi (1990), the first reading process generates a mental representation among readers, which can be activated during rereading to facilitate comprehension. Although the participants in our experiment were instructed to reread the text as a new article, natural variations in eye movement behavior still occurred. This indicates that readers had engaged in certain levels of cognitive processing of the text content after the first reading, integrating new context with their background knowledge to form new cognitive representations (van Moort et al., 2021). Therefore, during rereading, readers may have bypassed familiar information directly without excessive memory recall, reducing the reflection of momentary comprehension difficulties.

In addition to categorizing eye movements as forward saccades and regressions, we also developed a set of features specifically related to regressions. These features included regression counts and regression size within the line and across lines. These refined regression features can help capture the cognitive progressing characteristics of discourse reading.

Discourse comprehension is a multi-level processing task. Readers need to engage in inferential and integrative processes through the interaction of text information and background knowledge, thereby establishing effective and coherent mental models and memory representations (Carreiras and Cliftion, 2004; van den Broek, 2010; van Moort et al., 2021). This study showed that regression duration within the line and across lines was not useful in identifying rereading activities. Instead, rereading was primarily associated with the regression counts and regression size. This means that rereading effectively increased reading fluency in the later stages of the process of reading and comprehension (Xue et al., 2020). The regression of the first reading may be the need to obtain the necessary information to construct the context, mainly to search for information retrospectively on a large scale (Van Moort et al., 2021); while the regression in the rereading process is more of a goal-oriented active verification, and the reader may confirm or process the details in a targeted review on the background of the model that has been built. As such, rereading assists readers in more efficiently integrating the context and their background knowledge to comprehend the text, leading to the faster acquisition of relevant information. These findings are consistent with those of previous research (Abundis-Gutiérrez et al., 2018; Inhoff et al., 2019).

Furthermore, there was no significant difference in the pupil size and proportion of fixation duration between the first reading and rereading. This indicates that participants maintained a high level of attention during both reading sessions and successfully fixated on the text regions. It also reflects the effective control of fatigue effects in this experiment.

### 4.3 First reading-rereading differences in local eye movement parameters

There were significant differences in local eye movement behaviors between the first reading and rereading. When rereading, the first fixation duration, first-pass reading time, and first fixation counts were lower than during the first reading, which are early-stage parameters in reading processing. Consistent with previous research (Foster et al., 2013), rereading contributed to significant decreases in early processing measures (i.e., first fixation duration, gaze duration). This suggests that prior knowledge formed during the first reading can effectively assist readers in recognizing and understanding unfamiliar vocabulary upon rereading (Raney, 2003).

Furthermore, the participants had a significantly decreased total fixation duration, total number of fixations, regression out counts, selective regression path duration, second fixation duration, second-pass reading time, and second fixation counts when rereading. These eye movement indicators pertain to late-stage reading processing, which are typically associated with higher-level reading comprehension processing. Readers make connections between propositions in the text and their mental models. This involves a process of reanalysis following the encountering of reading difficulties (Or-Kan, 2017; Țurcan and Filik, 2016).

The results of this study indicate that rereading effectively reduces cognitive and attentional load. Local eye movement indicators support the dynamic processing view of the landscape model: rereading reuses the model constructed during the first reading and activates residuals, allowing resources to be used for active, precise processing in the later stages of reading, thereby significantly optimizing reading efficiency. Furthermore, this process is regulated by reading level. High-level readers, with their rich background knowledge and high consistency standards, are able to execute goal-oriented information integration more efficiently during rereading. In the late stage of reading processing, participants required less effort to read and comprehend the discourse. Therefore, readers’ information processing speed improved during rereading, and the time needed for information encoding and semantic extraction was shortened. In addition, rereading increased familiarity with the discourse material, enhanced anticipation of the text meaning, and improved information processing speed. Last, as mentioned, repetition and backward eye movements can assist readers in integrating and processing contextual information, which is related to deeper learning abilities such as the construction of readers’ mental models (Stanovich, 1980; van Moort et al., 2021).

### 4.4 Neural network classification model

The current study built a neural network classification model based on eye-tracking data. This model achieved an accuracy of 0.769, precision of 0.774, recall of 0.788, and F1-score of 0.781. The FI of local eye movement parameters is higher than of global eye movement parameters. The results of the experiment showed that eye movement behaviors are good predictors of first reading and rereading, particularly for local eye movement parameters.

Although previous eye-tracking prediction model studies achieved 96.2% accuracy in dyslexia detection (Nerušil et al., 2021), the criteria for distinguishing dyslexia are often independent and explicit. However, the detection of discourse rereading is more complex. Eye movement processes can be influenced by various factors such as text difficulty, reading proficiency, and test intervals (Callender and McDaniel, 2009; Greving and Richter, 2019; Xue et al., 2020), all of which can potentially decrease the performance of the neural network model’s detection. Therefore, for the classification of the first reading and rereading, our results showed a good predictive score. The classifier in this study outperformed the level of chance by a wide range. Furthermore, considering the previously mentioned application of neural network models in assessing reading proficiency, literacy levels, and dyslexia with eye-tracking (Copeland et al., 2014; Nerusil et al., 2022; Nerušil et al., 2021), eye-tracking technology has the potential for detecting the rereading effect in discourse comprehension and the evaluation of text familiarity.

This study contributes to differentiating the eye movement behavior of readers during the first reading and rereading, providing a deeper understanding of the cognitive mechanisms underlying the rereading effect on students. The neural network-based eye movement prediction model accurately predicts the first reading and rereading during discourse comprehension, offering empirical and technical support for related research (e.g., text familiarity). In terms of practical significance, this neural network model can help assess students’ current levels of reading comprehension and knowledge mastery, significantly improving the accuracy and efficiency of reading assessments. Furthermore, the prediction model can assist in optimizing learning material. Specifically, the global and local eye movement features of discourse rereading can be used to identify text paragraphs, sentences, or vocabulary information that students are prone to overlook or misunderstand, thereby optimizing instructional materials to better convey knowledge.

### 4.5 Limitations and further research

The current study has limitations that motivate future investigations: (1) This study only examined rereading effects in terms of total reading time and various eye movement parameters in university students. There are other aspects in which the rereading effect can be further explored, such as its impact on students’ cognitive effort, emotional arousal, and repeating the experiment on a larger population to validate the research conclusions. Additionally, the gender ratio in our sample was unbalanced (more females), constrained by recruitment patterns. While our analyses did not reveal significant gender effects on the observed rereading outcomes among adults as previous research (Nero and Zulkiply, 2020), future studies should recruit balanced samples to definitively assess potential gender influences. Furthermore, future research could explore the use of rereading strategies in real-world settings and the effects of rereading in everyday life. This will help improve the ecological validity and practical value of the research. For example, the research results can help teachers guide students in using rereading as a reading strategy to improve reading comprehension, or develop real-time electronic reading interaction and assessment software. (2) In this experiment, only one text material was utilized, which could be affected by the background knowledge of the students. The participants consisted of university students who are generally well educated. Future research should include multiple passages to provide a more balanced set of materials, based on assessments from professional teachers, and should be repeated across diverse demographic groups. (3) Neural network models that solely rely on knowledge-driven features as input variables tend to yield results that while easy to interpret, often have limited accuracy due to the restricted number of features included in the model. In addition, although the model may demonstrate a good fit on the training datasets, generalizing it to other datasets becomes challenging. Hence, future research could evaluate the eye movement for specific themes or areas, and future efforts could focus on combining knowledge-driven and data-driven approaches to explore more effective predictive indicators and construct neural network models that are more precise and stable.

## 5 Conclusion

Based on the previous results, the following conclusions were drawn: The eye movement parameters during digital discourse reading are applicable to classifying first reading and rereading, and the neural network model based on eye movement behaviors obtained convincing accuracy (76.9%) for the classification of rereading. Local eye movement parameters are more important for spotting rereading than overall eye movement parameters. These findings can provide anticipatory help to test the text familiarity of students.

## Data Availability

The datasets analyzed in this study are available at: https://doi.org/10.17605/OSF.IO/5A2UD.
