# Supplementary material for: Assessing the rereading effect of digital reading through eye movements using artificial neural networks
Source: Front Psychol. 2025 Aug 21;16:1576247. doi: 10.3389/fpsyg.2025.1576247 (PMC12409514; doi:10.3389/fpsyg.2025.1576247)
Supplement: Supplementary file 1 [file Supplementary_file_1.docx]

Supplementary Material

# Supplementary Figures

## Bar chart of regression parameters in global measures in the repeated measures analysis of variance (ANOVA)

##
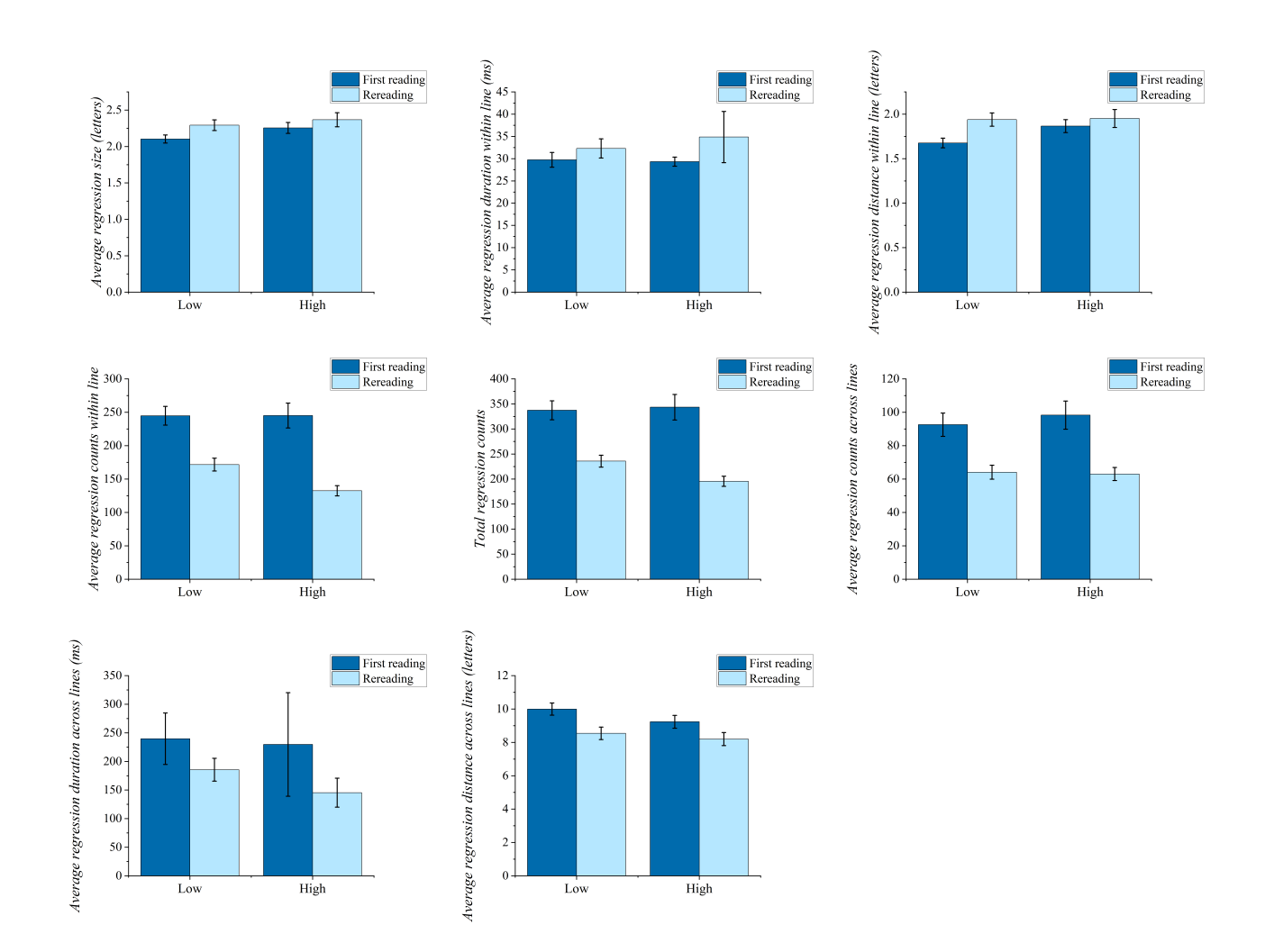


Fig. S1. Bar chart of regression parameters in global measures in the repeated measures analysis of variance (ANOVA)

Note: Error bar is constructed using one standard error from the mean.

## Bar chart of local eye movement parameters in the repeated measures analysis of variance (ANOVA)


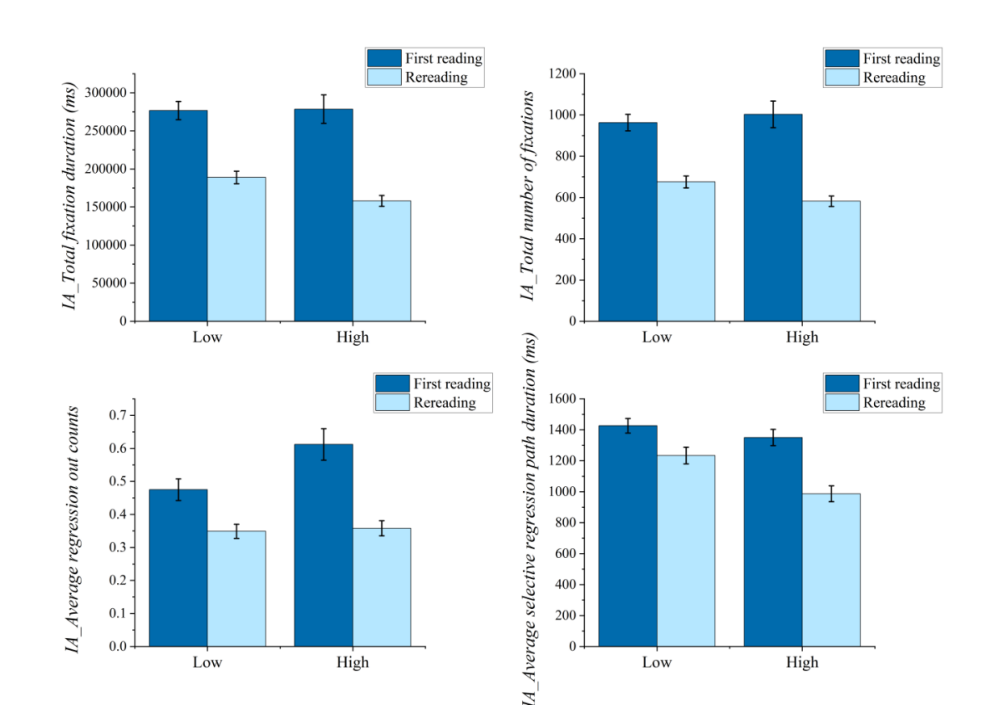


Fig. S2. Bar chart of local eye movement parameters in the repeated measures analysis of variance (ANOVA)

Note: Error bar is constructed using one standard error from the mean.

## Bar chart of the parameters of the first- and second-reading in local measures in the repeated measures analysis of variance (ANOVA)


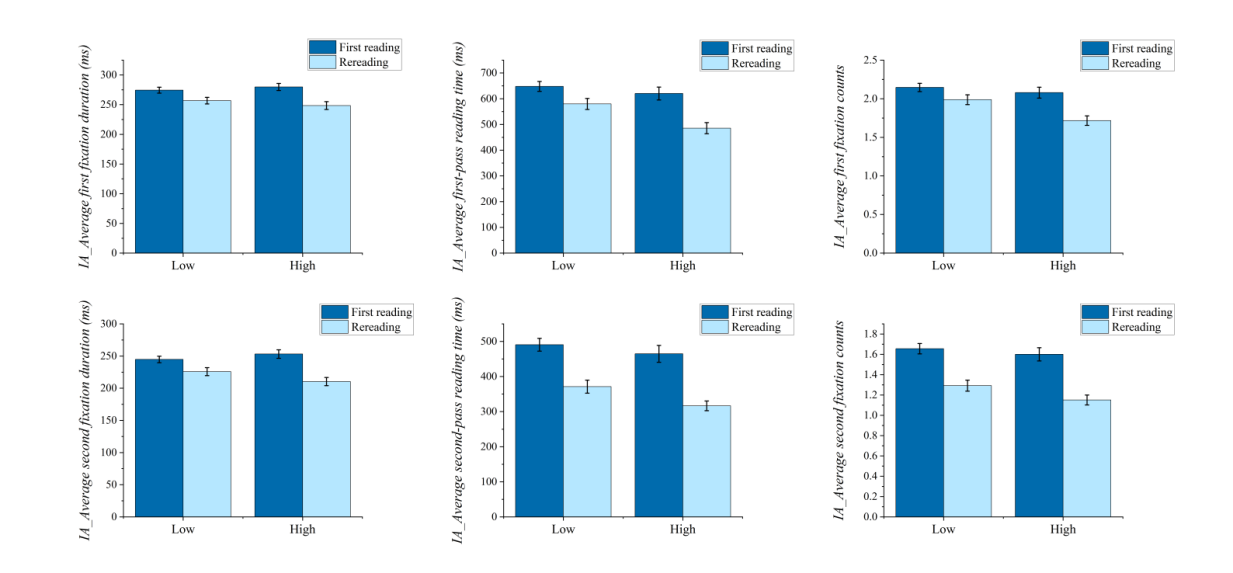


Fig. S3. Bar chart of the parameters of the first- and second-reading in local measures in the repeated measures analysis of variance (ANOVA)

Note: Error bar is constructed using one standard error from the mean.

## Final scores of loss and accuracy in the training and test set


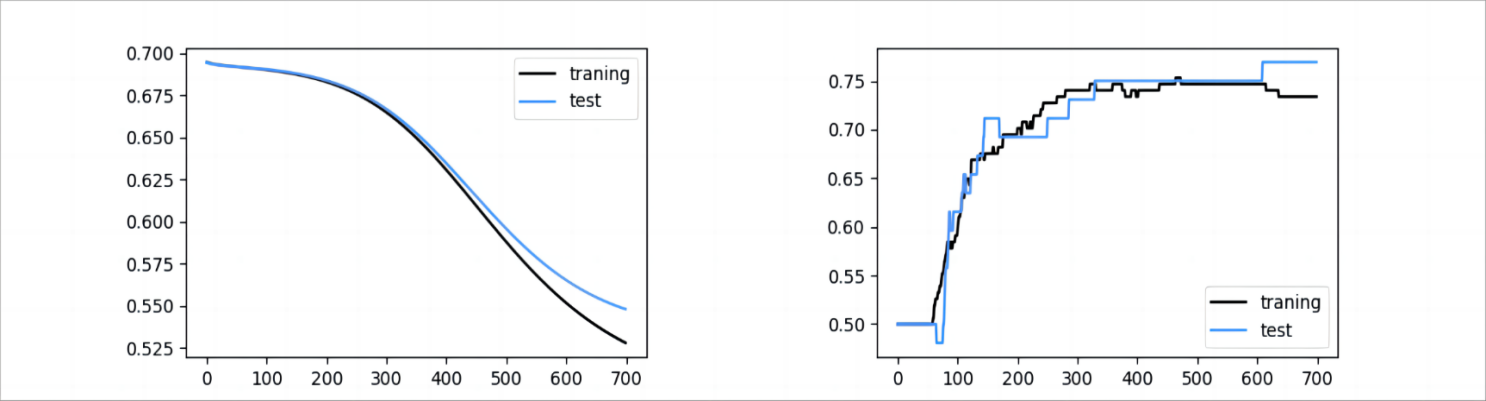
Fig. S4. Final scores of loss and accuracy in the training and test set

# Supplementary Tables

**2.1 Demographic Information of Participants**

Table S1 Descriptive Statistics of Population Variables and Reading Levels (N=103)

| **Variable** | **M** | **SD** | **Minimum** | **Maximum** |
| --- | --- | --- | --- | --- |
| Gender | 1.602 | 0.492 | / | / |
| Age | 23.165 | 2.997 | 18 | 32 |
| Grade | 5.194 | 2.368 | 1 | 13 |
| Major | 1.515 | 0.739 | / | / |
| CET-4 Score | 522.650 | 65.289 | 400 | 698 |
| CET-6 Score | 493.718 | 61.259 | 300 | 661 |

***Note:** Gender: 1 = Male, 2 = Female; Grade: 1-4 = Freshman to Senior, 5-7 = Master's Year 1 to Year 3, 8-12 = Doctoral Year 1 to Year 5, 13 = Other (Graduated); Major: 1 = Science, 2 = Engineering, 3 = Humanities.*

107 subjects were selected for this study, and four of them failed to collect data normally due to eye tracking technical problems. Finally, 103 subjects were included in the analysis. There were 41 males, accounting for 39.8% of the total. There were 62 females, accounting for 60.2% of the total. 65 students majored in science, accounting for 63.1%; 23 were engineering students, accounting for 22.3%; And 15 students from liberal arts, accounting for 14.6%. The age ranged from 18 to 32 years old (M±SD=23.17±3.00). The grade distribution was from freshman to doctoral fifth and other (graduation), of which 13 were senior students, accounting for 12.6%, 16 were graduate students, accounting for 15.5%, and 33 were graduate students, accounting for 32%, which were the three grades with the largest proportion.

**2.2 Descriptive Analyses of Participants’ Eye Movements**

The descriptive analyses of participants’ eye movements during the first reading and rereading, categorized by low- and high-level reading proficiency, are presented in Tables S2-5. The descriptive results in Table S2 and Fig. 2 show observable differences in the global eye movement parameters between the first reading and rereading. Table S3 and Fig. S1 (Fig. S1-S4 see supplementary materials) present the observable differences in the regression parameters in global measures between the first reading and rereading. Table S4 and Fig. S2 show the differences in local eye movement parameters between the first reading and rereading. Finally, Table S5 and Fig. S3 illustrate the differences between the parameters of the first- and second-reading in local measures.

Table S2 Descriptive statistics of the global eye movement parameters (*n*_1_=63, *n*_2_=40)

| **Eye movement**  **parameters** | **Reading proficiency** | **First reading (ms)** | | **Rereading (ms)** | |
| --- | --- | --- | --- | --- | --- |
|  |  | ***M*** | ***SD*** | ***M*** | ***SD*** |
| Total reading time | Low | 329074.0 | 111152.8 | 224282.7 | 72397.0 |
|  | High | 323734.9 | 137796.8 | 186453.3 | 51211.9 |
| Total number of fixations | Low | 962.7 | 316.9 | 675.5 | 229.6 |
|  | High | 1003.0 | 408.3 | 581.7 | 160.9 |
| Proportion of fixation duration | Low | 0.84 | 0.07 | 0.84 | 0.07 |
|  | High | 0.86 | 0.04 | 0.86 | 0.15 |
| Average fixation duration | Low | 287.8 | 30.4 | 281.0 | 31.9 |
|  | High | 278.6 | 33.2 | 272.9 | 34.5 |
| Total number of forward saccades | Low | 668.7 | 196.9 | 331.4 | 125.3 |
|  | High | 697.3 | 281.2 | 353.8 | 170.3 |
| Average pupil size | Low | 1255.6 | 311.0 | 1266.3 | 299.3 |
|  | High | 1299.5 | 363.5 | 1311.0 | 358.3 |

Table S3 Descriptive statistics of the regression parameters in global measures (*n*_1_=63, *n*_2_=40)

| **Eye movement**  **parameters** | **Reading proficiency** | **First reading (ms)** | | | **Rereading (ms)** | | |
| --- | --- | --- | --- | --- | --- | --- | --- |
|  |  | ***M*** | ***SD*** | | ***M*** | ***SD*** | |
| Total regression counts | Low | 337.349 | | 149.668 | 235.778 | | 94.371 |
|  | High | 343.475 | | 161.242 | 195.500 | | 64.018 |
| Average regression size | Low | 2.103 | | 0.440 | 2.293 | | 0.582 |
|  | High | 2.255 | | 0.473 | 2.368 | | 0.611 |
| Average regression duration within line | Low | 29.731 | | 13.218 | 32.294 | | 17.142 |
|  | High | 29.316 | | 6.450 | 34.855 | | 36.417 |
| Average regression distance within line | Low | 1.676 | | 0.429 | 1.939 | | 0.590 |
|  | High | 1.866 | | 0.461 | 1.951 | | 0.640 |
| Average regression counts within line | Low | 244.778 | | 111.548 | 171.667 | | 76.225 |
|  | High | 245.175 | | 117.454 | 132.475 | | 48.878 |
| Average regression counts across lines | Low | 92.571 | | 55.404 | 64.111 | | 33.0538 |
|  | High | 98.300 | | 53.389 | 63.025 | | 24.7246 |
| Average regression duration across lines | Low | 239.681 | | 356.722 | 185.456 | | 158.373 |
|  | High | 229.609 | | 572.493 | 145.373 | | 159.947 |
| Average regression distance across lines | Low | 9.994 | | 2.866 | 8.539 | | 2.972 |
|  | High | 9.234 | | 2.439 | 8.197 | | 2.485 |

Table S4 Descriptive statistics of the local eye movement parameters (*n*_1_=63, *n*_2_=40)

| **Eye movement**  **parameters** | **Reading proficiency** | **First reading (ms)** | | | **Rereading (ms)** | | |
| --- | --- | --- | --- | --- | --- | --- | --- |
|  |  | ***M*** | | ***SD*** | ***M*** | ***SD*** | |
| IA_Total fixation duration | Low | 276644.508 | 94695.630 | | 188821.190 | | 64616.970 |
|  | High | 278509.925 | 117905.585 | | 157987.975 | | 45294.460 |
| IA_Total number of fixations | Low | 962.698 | 316.921 | | 675.476 | | 229.623 |
|  | High | 1002.950 | 408.280 | | 581.675 | | 160.878 |
| IA_Average regression out counts | Low | 0.475 | 0.262 | | 0.349 | | 0.170 |
|  | High | 0.612 | 0.303 | | 0.358 | | 0.145 |
| IA_Average selective regression path duration | Low | 1426.668 | 373.751 | | 1233.756 | | 426.965 |
|  | High | 1350.487 | 331.346 | | 986.759 | | 324.370 |

Table S5 Descriptive statistics of the local eye movement parameters during the first- and second-reading (*n*_1_=63, *n*_2_=40)

| **Eye movement**  **parameters** | **Reading proficiency** | **First reading (ms)** | | | **Rereading (ms)** | | |
| --- | --- | --- | --- | --- | --- | --- | --- |
|  |  | ***M*** | | ***SD*** | ***M*** | ***SD*** | |
| IA_Average first fixation duration | Low | 274.436 | 40.208 | | 256.732 | | 43.921 |
|  | High | 279.826 | 37.505 | | 248.473 | | 41.800 |
| IA_Average first-pass reading time | Low | 647.811 | 153.496 | | 579.648 | | 169.525 |
|  | High | 620.233 | 159.571 | | 485.553 | | 137.126 |
| IA_Average first fixation counts | Low | 2.146 | 0.421 | | 1.988 | | 0.505 |
|  | High | 2.079 | 0.448 | | 1.717 | | 0.390 |
| IA_Average second fixation duration | Low | 244.792 | 39.877 | | 225.768 | | 48.730 |
|  | High | 253.167 | 41.731 | | 210.386 | | 39.905 |
| IA_Average second-pass reading time | Low | 490.663 | 145.727 | | 371.239 | | 146.616 |
|  | High | 464.772 | 151.822 | | 316.467 | | 87.177 |
| IA_Average second fixation counts | Low | 1.656 | 0.403 | | 1.292 | | 0.433 |
|  | High | 1.601 | 0.412 | | 1.151 | | 0.309 |

# Supplementary article and questions

Some people in the US have asserted that forgiving student loan debt is one way to stimulate the economy and give assistance to those in need. Some in US Congress have gone so far as to suggest forgiving up to $50,000 in debt per student borrower, but does forgiving student debt necessarily correlate to helping the economically disadvantaged?

The answer is no. This policy is just giving money away to universities and the most affluent students in attendance. Federal Reserve data reveals that the highest-income 40 percent of households owe approximately 60 percent of outstanding student debt, while the lowest 40 percent owe just under 20 percent. Plus, the majority of student debt is held by graduate degree earners, who earn approximately 25 percent more than their undergraduate counterparts. Clearly, giving free reign to banks to forgive student debt is a step in the wrong direction. Other proposals for broader, long-term student loan plans have some fundamental problems. Students will feel more comfortable making the irresponsible decision to go tens of thousands of dollars in debt to major in impractical or idealistic subjects if they know their loans will be forgiven.

This is especially concerning given the pandemic has rendered a college education practically worthless. Students are paying tens of thousands of dollars per year to live at home and be lectured on the Internet. Do we really want to tell colleges that they can get away with providing below-average service for an outrageous cost?

In the case of any of these student debt plans, working-class Americans who chose not to or could not afford to go to college will be subsidizing the education of the professional class. Plumbers and retail workers will be paying for the degrees of doctors and lawyers. The US government’s effort to help those in debt is commendable but is this really the solution that help the poor financially recover?

1. Why do some people advocate forgiving student loan debt?

A) They assert it will narrow the gap between the wealthy and the poor.

B) They believe it will benefit both the economy and the underprivileged.

C) They claim it will eliminate economic distress among college students.

D) They think the cost of education is the responsibility of the government.

2. What do we learn from the Federal Reserve data?

A) Approximately 60% of student debt remains unpaid.

B) Cancelling student debt benefits wealthy families most.

C) Forgiving student debt provides little benefit to universities.

D) Low-income families owe the biggest amount of student debt.

3. What does the author imply about colleges offering online education?

A) They cannot get away with the serious consequences.

B) They have suffered greatly from the current pandemic.

C) The tuition they charge is not justified by the quality of their service.

D) The tuition they charge has surged outrageously during the pandemic.

4. What will happen if any of the proposed student debt plans is implemented?

A) Plumbers and retail workers will have a chance of becoming professionals.

B) Working-class students will have increasing access to subsidized education.

C) Blue-collar workers will have to bear the cost of educating would-be high-earners.

D) A growing number of students will be able to earn degrees in medicine and law.
